# Influence of mentoring on the proactive behavior of new employees: moderated mediation effect of agreeableness

**DOI:** 10.3389/fpsyg.2024.1370815

**Published:** 2024-03-18

**Authors:** Wenjie Yang, Yuxue Wang, Myeongcheol Choi, Yannan Li

**Affiliations:** ^1^Business School, Lingnan Normal University, Zhanjiang, China; ^2^Guangdong Coastal Economic Belt Development Research Center, Zhanjiang, China; ^3^Department of Business, Gachon University, Seongnam, Gyeonggi, Republic of Korea; ^4^Graduate School of Technology Management, Kyung Hee University, Yongin, Republic of Korea

**Keywords:** mentoring, balanced psychological contract, proactive behavior, agreeableness, proactive motivation model

## Abstract

**Objective:**

In recent years, faced with a complex economic development environment and the evolving dynamics of the Chinese workplace, talent has become a precious resource that is invaluable yet scarce for every enterprise. As Generation Z employees have gradually entered the labor market, they contribute new perspectives and energies to various enterprises and pose unique challenges. The traditional step-by-step approach no longer meets the needs of today’s businesses. Companies require more proactive talents to drive superior performance. Individuals with proactive behavior can effectively plan their career paths and are better equipped to fulfill core organizational tasks. Therefore, it is crucial for organizations to effectively mitigate the perceived negative impacts of proactive behavior, encouraging individuals to exhibit more positive proactive actions.

**Methods:**

Based on the proactive motivation model, this study investigates the effects of mentoring, balanced psychological contract, proactive behavior, and agreeableness on the proactive behaviors of new employees. The research surveyed 417 new employees from Guangdong Province, China, who had graduated within the last three years, with a gender distribution of 49.4% male and 50.6% female.

**Results:**

Structural Equation Modeling was used for data analysis, and the following results were obtained: First, mentoring positively affected the balanced psychological contract and new employees’ proactive behavior. Second, mentoring positively affected the new employees’ proactive behavior through the balanced psychological contract. Third, agreeableness played a moderating role in the relationship between mentoring and new employees’ proactive behavior, and in the relationship between mentoring and the balanced psychological contracts. Finally, the positive indirect effect of mentoring through the balanced psychological contract on new employees’ proactive behavior is positively moderated by agreeableness.

**Conclusion:**

The results of this study offer new insights into mentoring research for new employees and provide practical guidance for fostering the balanced psychological contract and proactive behavior among new employees. This research enriches the existing literature on mentoring for new employees by demonstrating the integral roles of agreeableness and a balanced psychological contract in fostering proactive behavior, offering valuable insights for organizational practices aimed at enhancing employee proactivity.

## Introduction

Post-COVID-19, due to repeated changes in organizational needs and uncertainty in the work environment, unprecedented major changes have taken place in organizational strategy and management, and proactive behavior has become increasingly important (ALGaraawi and Rashid, 2023). Proactive behavior entails actively choosing to enhance oneself amidst the prevailing environment. Such behavior involves active engagement and challenge rather than mere passive adaptation (Crant, 2000) and can foster long-term positive development within organizational settings. However, it’s worth noting that in practicality, this proactive conduct carries an element of risk, as its outcomes may introduce an element of uncertainty (Morrison and Phelps, 1999), For example, failure in actions may result in defamation and damage to reputation (Ashford et al., 2003; Parker et al., 2010). Specifically, while most research highlights the potential positive outcomes of individual proactive behavior, there is also an acknowledgment of its costs, alongside analyses that reveal both its beneficial and detrimental effects on individuals and organizational contexts (Li and Huang, 2021). In this context, organizations enhance the requirement of employees’ proactive behavior. Managers expect employees to break the work limits, and independently identify, analyze, and solve problems, to help organizations resist external risks and maintain a competitive advantage (Riivari et al., 2020; Xu et al., 2021). Promoting proactive employee behavior emerges as a key solution to navigating the uncertainties and rapidly changing demands of modern work environments, enhancing job satisfaction and mitigating negative outcomes (ALGaraawi and Rashid, 2023). The process of mentoring provided valuable training for the development and adaptation of new employees. Unlike coaching, which emphasizes short-term attention to specific aspects of an individual’s work, mentoring focuses on the long-term impact and comprehensive career development of the mentee (Clutterbuck, 2008; Stokes et al., 2021). The significant role of mentors is manifested in assisting new employees in acquiring the necessary knowledge, skills, and understanding of work dynamics (Zeng et al., 2020). Furthermore, mentors not only offer guidance at a professional level but also provide psychological support to young people (new employees), serving as role models (Kram and Isabella, 1985). This support promotes the smooth integration of new employees into their respective professional realms and fosters their career development (Kram and Isabella, 1985; Zehra et al., 2023). Therefore, Mentoring is crucial for organizations that intend to gain an advantage in a complex market environment (Allen and Eby, 2007; Scandura and Pellegrini, 2007).

In the United States, over one-third of the large firms have implemented mentoring, and this number is increasing annually. However, little is known about mentoring in Asian countries such as China (Zhou et al., 2019). In China, the corporate mentoring system has been invoked since the 1990s, but has been neglected by many organizations that have overlooked the value of the mentoring. The reason for this may be that mentors are worried about their protégés surpassing themselves with the help of their own resources, and thus choose to retain the efficiency of mentoring (Zeng et al., 2021). Moreover-most studies on employee proactive behavior in China focus on leadership style, and there are fewer studies on mentoring behavior. Therefore, it is worth exploring how new employees can be given more mentoring and through what mechanisms they can be consolidated to show more proactive behavior towards the organization? This study proposes the following research questions: (1) Whether mentoring has an effect on the proactive behaviors of new employees? (2) Are there some kind of mediating mechanism for such an effect? and (3) Are there individual differences involved?

According to the proactive motivation model by Parker et al. (2010), to motivate someone to pursue their goals actively, they need to be in a state of “can do,” “reason to,” or “energetic to.” From the standpoint of individual variances, the person-organization fit theory posits personal strengths and resilience as pivotal components (Kristof, 1996). Within this theoretical framework, an imperative lies in expanding research of personality dimensions and individual dissimilarities, particularly concerning the context of thriving organizations and resilient, flourishing employees (Di Fabio, 2017). However, there has been limited exploration into the outcomes of mentoring functions from the perspective of individual differences, representing a significant area for further study (Banerjee-Batist et al., 2019). Considering the scope of the Big Five personality traits, agreeableness assumes a noteworthy role in shaping an individual’s emotional inclinations, where elevated levels of agreeableness are correlated with heightened positive emotional responsiveness (Tobin et al., 2000). The balanced psychological contract reflects the positive emotional side of the individual (Rousseau, 2001). Recent studies highlight the pivotal role of mentoring in enhancing proactive behavior among newcomers, illustrating that effective mentorship significantly aligns employee efforts with organizational goals (Wu et al., 2019). Additionally, research identifies the balanced psychological contract as a crucial mediator in the dynamic between inclusive leadership and proactive work behavior, emphasizing that the fulfillment of psychological contracts is vital for promoting proactivity within the workforce (Rogozińska-Pawełczyk, 2023). Moreover, the trait of agreeableness in managers, which fosters greater employee interaction, trust-based relationships, and a responsive attitude towards staff needs, is shown to encourage the formation of more relational psychological contracts, thereby facilitating proactive behaviors among employees (Metz et al., 2017).

This study is designed to conduct a detailed exploration of the influence of proactive behavior in newly hired employees, especially focusing on the influence of mentoring in promoting proactive behavior, the mediating role of the balanced psychological contract in this relationship, and the moderating effect of agreeableness. By synthesizing a comprehensive review of current academic literature with empirical research, this investigation seeks to dissect the interplay among these elements and their combined impact on the proactive behaviors of new hires. The objective is to uncover insights into how organizations can foster such behaviors, thereby enabling employees to more effectively navigate the complexities of an evolving development environment, which in turn facilitates the attainment of corporate sustainability.

## Theory and hypotheses

### Mentoring and proactive behavior

Mentoring has three functions: career support, psychological support, and role modeling. These functions can assist employees in terms of career exposure and protection, increased psychological identity and self-efficacy, and role-modeling actions (Kram and Isabella, 1985; Scandura and Ragins, 1993; Allen et al., 2017). Additionally, mentoring correlates better with proactive behavior (Wu et al., 2019), motivation, and attitudes, especially in the workplace (Eby et al., 2008). First, career support can be provided to new employees via exposure. Specifically, career support from a mentor can introduce new employees to more people within the organization and improve their interpersonal communication and sense of belonging (Kram and Isabella, 1985; Choi and Yu, 2022). These can give employees the energy to not worry about the negative consequences of exhibiting proactive behavior and the negative consequences of making mistakes, which is one of the prerequisites for proactive behavior (Wörtler et al., 2020).

Second, in terms of psychological support, mentoring can enhance the identity, self-efficacy, and personal values of employees (Allen et al., 2017). Self-efficacy is a motivating factor of proactive behavior (Frese and Fay, 2001). Moreover, people with higher self-efficacy are more confident in helping others remove obstacles and engage in proactive behaviors toward goals (Bandura, 1997; Parker et al., 2006). In addition to self-efficacy, new employees’ self-values can also impact their proactive behaviors (Martin, 2016). The mentoring can contribute to a mentee’s career growth, demonstrate an environment of development and self-worth in the organization, and increase employees’ identification with the organization (Chen and Wen, 2016), which in turn is a factor that positively influences employees’ proactive behaviors (Etodike et al., 2020).

Finally, role models refer to the power of role models, which can enhance mentee’s self-esteem (Allen et al., 2004). When a new employee encounters a mentor with the power of role modeling, it accelerates the clarification of the employee’s role in the organization and facilitates faster and more positive integration into the organization (Kozák and Krajcsák, 2018). Researchers have confirmed that higher levels of mentoring can increase mentees’ self-esteem in the organization, which promotes positive proactive behaviors (Wu et al., 2019). Based on this, the following hypothesis is formulated:

*H1*: Mentoring is positively related to new employees’ proactive behavior.

### Mentoring and balanced psychological contract

The psychological contract is an implicit, reciprocal, desired, and critical core agreement between individuals and organizations (Herriot et al., 1997). Its positive (balanced) state determines an individual’s level of personal fulfillment and commitment to the organization, as well as the level of benefit to the organizations (Wellin, 2016). Different psychological contracts are formed by new employees based on the information they observe or feel from their organization, and their different types of psychological contracts are relatively stable and long-lasting (Rousseau, 2001).

Rousseau (1995) discussed the importance of mentors in the formation and evaluation of psychological contracts. Mentoring and psychological contracts are related and important organizationally-based social exchange relationships (Haggard, 2012). When an organization provides professional and psychological support and care to employees, the employees also exhibit their true psychological feelings and behavior toward the organization (O’Donohue et al., 2018). So when the more the organization cares for its employees, the more likely they are to form a sense of dependence on the organization, and in turn employees may form higher emotional commitment as well as attachment to the organization. Mentoring can professionally, psychologically, and role-wise strengthen employees’ emotional commitment (Allen et al., 2004). When mentoring is higher, employees form a deeper attachment to the organization and are more likely to promote a balanced psychological contract to balance interpersonal interactions and relationships with the organization (Ntalianis and Dyer, 2021). Based on this, the following hypothesis is proposed:

*H2*: Mentoring is positively related to balanced psychological contract.

### The mediating role of the balanced psychological contract

Psychological contracts can explain the relationship between individuals and organizations, and their creation can explain employees’ work attitudes and behaviors (Conway and Briner, 2002). The changes induced by the different psychological contracts of employees mainly emanate from the organization’s behavior toward them, which, in turn, impacts employees’ attitudes and behaviors toward the organization (O’Donohue et al., 2018). This indicates that when the organization’s behavior and purpose towards the employees is such that the employees feel positive or satisfied, then their interpersonal relationships and motivation will also be better displayed in order to balance the organization. When individuals experience positive affective states, they are more likely to promote a balanced psychological contract and show higher proactive behaviors, whereas negative affective states induce lower proactive behaviors (Bal et al., 2011; Parker and Bindl, 2017).

For new employees, proactivity can be facilitated by paying attention to their proactive collection of information about different psychological contracts at the initial stage of their induction (De Vos et al., 2005). In this case, the more guidance a mentor provides to a new employee, the more information the employee collects about the different psychological contracts (De Vos and Freese, 2011), and the higher their initiative.

We can conclude that when employees have a psychological contract construct, they exhibit more proactive behaviors toward the organization. This is because mentoring provides them with support and demonstrates the organization’s emotional care, which promotes a balanced psychological contract state. Based on this, the following hypothesis is proposed:

*H3*: Balanced psychological contract mediates the positive relationship between Mentoring and proactive behavior.

### Moderated mediation effect of agreeableness

Personality refers to the natural cognitive responses and emotional patterns that individuals develop owing to environmental factors (Corr and Matthews, 2020). Parker et al. (2010), in their study “A model of proactive motivation, “elucidate how proactive behavior, defined as a goal-driven process including proactive goal generation and striving, is supported by “can do,” “reason to,” and “energized to” motivational states. These states, influenced by individual differences and the surrounding context, significantly complement our comprehension of how the trait of agreeableness not only predisposes individuals to altruistic and proactive helping behaviors but also interacts with environmental and interpersonal dynamics to promote proactive behaviors in organizational contexts.

Agreeableness reflects an individual’s ability to be warm, kind, helpful, honest, and considerate toward people and events (Rothmann and Coetzer, 2003; Thompson, 2008; Graziano and Tobin, 2009). In terms of behavior, people with agreeable personalities have an innate tendency to actively help others (Penner et al., 1995). This is the result of the altruistic component of this personality type, which encourages people to be more active in their proactive helping behaviors, sometimes without any external motivational factor (Graziano et al., 2007). For example, individuals with high levels of agreeableness are more likely to exhibit active organizational citizenship behaviors (Guay et al., 2013). Additionally, they may be more willing to actively exhibit sharing behaviors (Anwar, 2017). Moreover, organizational commitment increases with high agreeableness, and employees exhibit more positive proactive behaviors (Strauss et al., 2009; Guay et al., 2016). The “five virtues” of Chinese Confucianism, which represent kindness and goodness, fairness and justice, courtesy and politeness, wisdom and intelligence, and loyalty and honesty, are more similar to the traits of agreeableness. Because the “five virtues” of Chinese Confucianism have been the values of the Chinese people, and these values influence individual behavior and emotions (Kang et al., 2017). In addition, different personalities of individuals are capable of influencing the degree of effectiveness of the mentoring (Engstrom, 2016). In other words, individuals with high levels of agreeableness are better at accepting the impact of mentoring and thus influencing their own behavior.

In terms of interpersonal relationships, individuals with agreeable personalities have exceptional interpersonal relations in groups and are adept at regulating or balancing conflicts in the group (Graziano et al., 2007). This suggests that such people are highly altruistic, prioritize the interests of the organization and others, and always have an optimistic outlook toward people and situations. Given their ability to be more sensitive to the positions, motivations, and perspectives of others (Graziano et al., 2007), agreeable individuals easily adjust to and are recognized by others (Song and Shi, 2017; Bamford and Davidson, 2019). This further suggests that agreeableness enables individuals to maintain good mental health and positive relationships with others. Additionally, among the big five personality traits, agreeableness can influence an individual’s emotional tendencies, and the higher the agreeableness, the stronger the positive emotional response (Tobin et al., 2000). This primarily manifests in the individual’s self-control during the emergence of negative emotions (Jensen-Campbell and Graziano, 2001), thereby avoiding the display of less proactive behaviors (Bal et al., 2011; Parker and Bindl, 2017). Therefore, while agreeableness encompasses a broader range of interpersonal attributes, its altruistic component is particularly relevant to understanding proactive behaviors in organizational settings.

In terms of mentoring, the effectiveness of mentoring is more effective when the mentor’s experience or personality is similar to that of the apprentice (Engstrom, 2016; Humberd and Rouse, 2016; Zhou et al., 2019). Moreover, coupled with the fact that agreeableness individuals are susceptible to others’ influence, their performance is better when others have a positive influence; conversely, when easygoing individuals receive negative influences, it can lead to extremely bad behavior (Walters, 2018). According to attachment theory, agreeableness is a major predictor of secure attachment (Deniz, 2011), and career support in the mentoring provides just enough to safeguard this sense of security (Kram and Isabella, 1985). So when new employees have a high level of agreeableness, mentoring induces in them more altruistic factor and an increased willingness to accept the benefits of the mentoring. These benefits enhance employees’ self-efficacy, values, and identity, indirectly influencing their subsequent proactive behaviors (Chen and Wen, 2016; Hong et al., 2016; Etodike et al., 2020).

According to the proactive motivation model the interaction term E (individual differences such as personality) x F (strength of support such as leadership) may trigger proactive motivation mechanisms. Thus, a more agreeable personality enables new employees who have received mentoring to show more proactive behaviors and promote the emergence of positive affect in the balanced psychological contract. Based on this, the following hypotheses are proposed:

*H4*: Agreeableness moderates the positive relation between mentoring and proactive behaviors.

*H5*: Agreeableness moderates the positive relation between mentoring and balanced psychological contract.

*H6*: Agreeableness moderates the positive relation between mentoring through balanced psychological contract and proactive behaviors such that the relationship is strengthened when Agreeableness is high.

**Figure 1:**
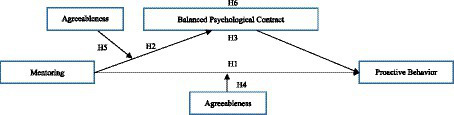
Research model.

The research hypothesis can be summarized as shown in Figure 1, which illustrates the research model.

## Methods

### Sample and data collection

Based on the proactive motivation model, the data collection for this study commenced in March 2021, with samples exclusively drawn from newly employed individuals in Guangdong Province, China, who have recently graduated from undergraduate programs and have less than 3 years of work experience. First, because of the global epidemic, the questionnaire needed to be sent through a combination of online and offline, so this study proceeded to form a Chinese WeChat group. Second, 20 university teachers specializing in tracking employment in Guangdong Province, China were invited, and each teacher was asked to randomly recommend about 30–40 undergraduate students who had just graduated and joined the workforce within 3 years as survey respondents. With the full support of 20 teachers, 15 WeChat groups have been formed according to different colleges, with a total of 500 people.

A description of the research study was subsequently conducted with 500 people from each of the 15 WeChat groups. Firstly, clearly state the purpose of the investigation, informing all participants that the data obtained will be used for scientific research, ensuring absolute confidentiality of the information involved, and guaranteeing that no negative impacts will result on their work or personal lives etc. Secondly, we gave simple instructions for filling out the questionnaire on mentoring, asking new employees to recall the person who has given them the most guidance since graduation, which could be a leader, a supervisor, or a coworker. For the other variables, the questions were filled out in such a way as to allow the participants to understand the meaning of the questions as much as possible without influencing or inducing them to do so. Thirdly, in order to increase the completion rate and efficiency of the questionnaire, this study gives each participant a reward of 15 RMB after completing the questionnaire. Moreover, to prevent multiple submissions, the survey is set to allow only one response per ID. Finally, after pilot testing, with the help of teachers, and through continuous efforts, persistence and contact, 417 valid questionnaires were finally obtained. The gender distribution within the sample is nearly balanced, the respondent sample profiles are summarized in Table 1.

**Table 1:** Descriptive analysis of participant.

**Demographic variable**	**Type**	**Frequency**	**Ratio (%)**
Gender	Male	206	49.4
	Female	211	50.6
Tenure	Less 1 Year	280	67.1
	1–2 Year	125	30
	2–3 Year	12	2.9
Job transitions	First job	333	79.9
	Second job	74	17.7
	Third job	9	2.2
Nature of organizations	Public institutions	160	38.4
	State-owned enterprises	23	5.5
	Private enterprises	147	35.3
	Joint venture units	5	1.1
	Government units	12	2.9
	Units	70	16.8
Total		417	100

### Measures

All variables in this study were measured using a five-point Likert scale. The mentoring in this study was based on the scale developed by Castro et al. (2004), which has good reliability in terms of gender and cross-cultural aspects (e.g., “I will try to follow the example of my mentor in the workplace”) (Hu, 2008; Hu et al., 2011). The proactive behaviors were based on the scale developed by Griffin et al. (2007), which is a self-reported (e.g., “I will create a better way to do my important work”). The balanced psychological contract was adopted from the psychological contract inventory developed by Rousseau (2000). The topics of balanced psychological contracts in this inventory are all self-subjective assessments (e.g., “I will actively seek internal training and development opportunities”). The 15-question big five personality scale developed by Zhang et al. (2019). was used to measure agreeableness, which was validated for the Chinese context, with five personality dimensions and three questions per personality dimension (e.g., “I feel that most people are basically well-intentioned”).

In the conducted study, the software utilized included SPSS Statistics 25 for data analysis, while AMOS 24 was employed for structural equation modeling. The analysis process followed a systematic approach. Initially, an examination of the demographic characteristics of the sample was carried out, encompassing frequency and ratio determination. Subsequently, the reliability of measurement tools was assessed through the calculation of Cronbach’s α coefficient, which employed dot product consistency analysis. Moreover, an evaluation of the variables’ discriminant and convergent validity was conducted, involving feasibility confirmation through factor analysis. Furthermore, the study involved a correlation analysis to assess the interrelationship between variables. To test the hypotheses, a path analysis was performed using AMOS. Finally, the research model was scrutinized through the lens of the theoretical framework proposed by Baron and Kenny (1986), following a stepwise process to verify the presence of mediating effects.

## Results

### Descriptive analysis and correlations

In this study, we utilized SPSS 25 to conduct descriptive statistics and Cronbach’s α tests on the data for each variable. The basic descriptive statistics and correlations of the measures are concisely summarized in Table 2. Our analysis indicates that all examined relationships among new employees’ proactive behavior, balanced psychological contract, mentoring, and agreeableness exhibit statistically significant correlations. These findings lay the groundwork for the subsequent testing of our research model and hypotheses, eliminating the need for an explicit repetition of each relationship’s positive significance in the text.

**Table 2:** Descriptive analysis, correlations and discriminant validity.

**Variables**	**Mean**	**SD**	**M**	**BPC**	**PB**	**AN**
Mentoring	3.282	0.901	1			
Balanced psychological contract	3.766	0.710	0.225**	1		
Proactive behavior	3.747	0.559	0.236**	0.396**	1	
Agreeableness	3.793	0.646	0.275**	0.290**	0.240**	1

*N* = 417. *p < 0.05, **p < 0.01 (two-tailed). M, mentoring; PB, proactive behavior; BPC, balanced psycho-logical contract; AN, agreeableness.

### Reliability, validity, and common method bias

To assess the measurement reliability and validity, we conducted a confirmatory factor analysis (CFA) using AMOS 24. The CFA results are presented in Table 3. As shown in Table 3, the model provides a good fit to the data [*χ*^2^ = 558.143, x^2^/DF = 3.624, *p* < 0.01, comparative fit index (CFI) = 0.935, Normed Fitness Index (NFI) = 0.913, incremental fit index (IFI) = 0.936, root mean square error of approximation (RMSEA) = 0.079, standardized root mean square residua (SRMR) = 0.063] (Anderson and Gerbing, 1988). Furthermore, all the factor loadings are highly significant (*p* < 0.001), and both the coefficient alpha values (0.880–0.931) and the composite reliabilities (0.882–0.926) of all the constructs exceed the 0.70 benchmark. All the average variances extracted (AVE) are >0.50. Therefore, our measures demonstrate adequate convergent validity and reliability (Fornell and Larcker, 1981). To assess discriminant validity, this research follow Fornell and Larcker’s (1981) procedure to compare the shared variance between all the possible pairs of constructs to determine whether they are lower than the AVE of individual constructs. A one-way confirmatory factor analysis (CFA) was used to test the variables for common method bias (Kline, 2013), and the results of the one-factor model are as follows. *x*^2^ = 3722.193, *x*^2^/DF = 23.558, CFI = 0.431, NFI = 0.421, IFI = 0.432, SRMR = 0.193, RMSEA = 0.233, thus indicating that there is no common method bias problem in the data of this study.

**Table 3:** Results of confirmatory factor analysis.

**Variables**	**Items**	**Estimate**	**AVE**	**CR**	**α**
Mentoring	M1	0.955	0.809	0.926	0.931
	M2	0.968			
	M3	0.761			
Balanced psychological contract	BPC1	0.847	0.710	0.907	0.905
	BPC2	0.881			
	BPC3	0.799			
	BPC4	0.832			
Proactive behavior	PB1	0.881	0.714	0.882	0.880
	PB2	0.810			
	PB3	0.842			
Agreeableness	AN1	0.846	0.763	0.906	0.905
	AN2	0,871			
	AN3	0.902			

### Direct effect and mediation analysis

SEM analysis was conducted to calculate the relationships among focus variables with gender and grade being controlled and to conduct mediation analysis. The results were tested using path analysis, as shown in Table 4. H1 and H2 were supported by the data as mentoring positively and significantly influences the psychological contract (*β* = 0.126, C.R. = 2.970, *p* = 0.003) and proactive behavior (*β* = 0.086, C.R. = 2.718, *p* = 0.007).

**Table 4:** The results of hypothesis testing(H1 and H2).

**Path**	**Estimate**	**S.E.**	**C.R.**	**P**
Mentoring → Balanced psychological contract	0.126	0.042	2.970	0.003
Mentoring → Proactive behavior	0.086	0.032	2.718	0.007

*N* = 417. *p < 0.05. **p < 0.01 (two-tailed).

According to the mediation effect test method proposed by Baron and Kenny (1986), as shown in Table 5, first, the independent variable needs to significantly affect the dependent variable, and it can be seen from Model 3 that the independent variable has a mentoring (*B* = 0.148, SE = 0.030, *p* < 0.001), and positively affects the dependent variable new employees’ proactive behavior. Secondly, the independent variable is required to significantly positively affect the mediator variable. From Model 2, it can be seen that the independent variable mentoring (*B* = 0.176, SE = 0.038, *p* < 0.001) significantly positively affects the mediator variable balanced psychological contract. Finally, when Model 4 controls the mediator variable balanced psychological contract, the positive effect of mentoring (*B* = 0.098, SE = 0.029, *p* < 0.01) on new employee’s proactive behavior is significantly reduced. At the same time, the mediator variable balanced psychological contract (*B* = 0.285, SE = 0.036, *p* < 0.001) has a significant positive effect on new employee’s proactive behavior.

**Table 5:** Mediating effects of balanced psychological contract.

**Variables**	**Balanced psychological contract**				**Proactive behavior**			
	Model 1		Model 2		Model 3		Model 4	
	*B*	SE	*B*	SE	*B*	SE	*B*	SE
Gender	0.000	0.070	−0.006	0.068	0.073	0.053	0.074	0.050
Job transitions	−0.061	0.072	−0.011	0.071	0.047	0.056	0.050	0.052
Tenure	−0.021	0.065	−0.003	0.064	−0.035	0.050	−0.034	0.047
Mentoring			0.176***	0.038	0.148***	0.030	0.098**	0.029
Balanced psychological contract							0.285***	0.036
*R*^2^	0.002		0.051		0.062		0.186	
Δ*R*^2^	−0.005		0.042		0.053		0.177	
*F*	0.280		5.506		6.856		18.839	

*N* = 417. *p < 0.05, **p < 0.01, ***p < 0.001 (two-tailed).

Table 6 shows that the mediating effect of psychological contract between mentoring and new employees’ proactive behavior is 0.040. This indicates an effective mediating effect of psychological contract on the relationship between mentoring and new employees’ proactive behavior. Therefore, Hypothesis 3 was supported.

**Table 6:** Mediating effects of balanced psychological contract.

**Parameter**	**Estimate**	**Product of coef.**		**Bias-corrected**		**Percentile**	
		SE	C.R.	Lower	Upper	Lower	Upper
Total	0.126	0.034	3.706	0.060	0.193	0.059	0.191
Direct	0.086	0.033	2.606	0.021	0.149	0.020	0.148
Indirect	0.040	0.016	2.500	0.011	0.076	0.010	0.074

### Mediation analysis

The moderating variable is considered to have a moderating effect when the interaction term of the product between the independent and moderating variables significantly affects the dependent variable (Busemeyer and Jones, 1983; Hayes and Rockwood, 2020).

**Figure 2:**
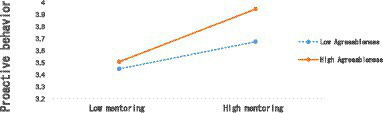
The moderating effects of agreeableness 1.

**Figure 3:**
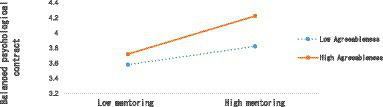
The moderating effects of agreeableness 2.

Table 7 shows that in Model 1, agreeableness has a significant positive effect on the balanced psychological contract of new employees (*B* = 0.272, SE = 0.053, *p* < 0.001). In Model 2, the interaction term (mentoring × agreeableness) has a significant positive effect on the balanced psychological contract of new employees (*B* = 0.130, SE = 0.051, *p* < 0.05) In Model 3, agreeableness has a significant positive effect on the balanced psychological contract of new employees (*B* = 0.167, SE = 0.042, *p* < 0.001). In Model 4, the interaction term (mentoring × agreeableness) has a significant positive effect on the proactive behavior of new employees (*B* = 0.106, SE = 0.040, *p* < 0.01), indicating that Hypotheses 4 and Hypotheses 5 was supported. This study further tested the moderating variables by plotting the moderating effects (Aiken et al., 1991; Dawson, 2014; Fang et al., 2015). Figures 2, 3 revealed the results.

**Table 7:** Moderating test of agreeableness.

**Variables**	**Balanced psychological contract**				**Proactive behavior**			
	Model 1		Model 2		Model 3		Model 4	
	*B*	SE	*B*	SE	*B*	SE	*B*	SE
Gender	−0.005	0.066	−0.001	0.066	0.074	0.052	0.077	0.052
Job transitions	0.016	0.070	0.023	0.069	0.063	0.055	0.070	0.055
Tenure	0.001	0.062	0.009	0.062	−0.033	0.049	−0.026	0.049
Mentoring	0.125**	0.039	0.121**	0.038	0.117***	0.031	0.113***	0.030
Agreeableness	0.272***	0.053	0.270***	0.053	0.167***	0.042	0.166***	0.042
Mentoring × Agreeableness			0.130*	0.051			0.106**	0.040
*R*^2^	0.107		0.121		0.097		0.112	
Δ*R*^2^	0.096		0.108		0.086		0.099	
*F*	9.840		9.384		8.791		8.579	

*N* = 417. *p < 0.05, **p < 0.01, ***p < 0.001 (two-tailed).

Referring to Table 8, upon integrating the moderator variable of agreeableness and the interaction term (mentoring × agreeableness) into Model 3, the mediating effect of balanced psychological contract (*B* = 0.255, SE = 0.037, *p* < 0.001). This outcome highlights the role of agreeableness in positively moderating effect of the balanced psychological contract on the relationship between mentoring and new employee’s proactive behavior. Thus, there’s preliminary support for Hypothesis 6.

**Table 8:** The result of moderated mediation effect (Hypotheses 5).

**Variables**	**Proactive behavior**					
	Model 1		Model 2		Model 3	
	*B*	SE	*B*	SE	*B*	SE
Gender	0.073	0.053	0.077	0.052	0.077	0.049
Job transitions	0.047	0.056	0.070	0.055	0.064	0.052
work experience	−0.035	0.050	−0.026	0.049	−0.029	0.046
Mentoring	0.148***	0.030	0.113***	0.030	0.082**	0.029
Agreeableness			0.166***	0.042	0.097*	0.041
Mentoring × Agreeableness			0.106**	0.040	0.073	0.039
Balanced psychological contract					0.255***	0.037
*R*^2^	0.062		0.112		0.204	
Δ*R*^2^	0.053		0.099		0.190	
*F*	6.856		8.579		14.964	

*N* = 417. *p < 0.05, **p < 0.01, ***p < 0.001 (two-tailed).

In order to probe into the moderated mediation of agreeableness, this study applies the approach advocated by Hayes (2013). Using SPSS 25 software, 5,000 Bootstrap tests are executed, with a 95% confidence interval. The results of Bootstrap test are shown in Table 9. As discerned from Table 9, it becomes apparent that at a low value of 0.010 (LLCI = -0.026, ULCI = 0.047, encompassing 0) of agreeableness, there is not substantial adjustment to the mediation path connecting the mentoring of the balanced psychological contract and the proactive behavior of new employees. However, at the moderate value of 0.034 (LLCI = 0.010, ULCI = 0.060, excluding 0) and the high value of 0.057 (LLCI = 0.026, ULCI = 0.095, excluding 0), agreeableness positively adjusts the mediation pathways linking the mentoring of balanced psychological contract and new employee’s proactive behavior. This observation underscores the varying impact of agreeable personality levels on the relationship between the mentoring and new employees’ proactive behavior via the medium of the balanced psychological contract. Hypothesis 6 was supported.

**Table 9:** Bootstrap test results.

**Agreeableness**	Mentoring ➔ Balanced psychological contract➔ Proactive behavior			
	*B*	Boot SE	Boot LLCI	Boot ULCI
Effect 1(M-1SD)	0.010	0.018	−0.026	0.047
Effect 2(M)	0.034	0.013	0.010	0.060
Effect 3(M + 1SD)	0.057	0.018	0.026	0.095

95% confidence interval levels; Number of bootstrap samples for percentile bootstrap confidence intervals: 5,000. +/− SD from the mean.

## Discussion

This study assessed the direct, mediating, and moderating effects of the mentoring on the proactive behavior of new employees using the proactive motivation model developed by Parker et al. (2010). This empirical study achieved its research objectives and made significant contributions to the literature.

Firstly, this research demonstrated the positive effect of mentoring on new employees’ proactive behavior. It not only confirms that mentoring can promote new employees’ proactive behavior, but also supports the question raised by Parker et al. (2006): The presence of mentoring can motivate mentees to proactive behavior. However, the risks associated with proactive behavior, such as the potential for failure, cannot be ignored. As previously mentioned, actions that lead to failure may result in negative outcomes, including being slandered by others and suffering damage to one’s reputation (Ashford et al., 2003; Parker et al., 2010). Based on this, organizations can consider models such as reward policies to increase motivation of mentors (Chen et al., 2014; Janssen et al., 2018), and promote proactive behaviors among employees.

Secondly, the mediating effect of the balanced psychological contract was confirmed in this study. This complements the F-motivation line mechanism of the “hot” state “energetic to” in the proactive motivation model proposed by Parker et al. (2010). This study not only contributes to the balanced psychological contract theory, but also verifies the moderating of agreeableness. Chang and Uen (2022) argued that a mentoring system and positive personality traits positively affect organizational performance. New employees receive varied information that is beneficial to them and enables the fulfillment of different types of psychological contracts. In other words, for new employees, the higher the support of the mentoring in the organization, the more likely they are to fulfill a balanced psychological contract. Whereas, employees with a balanced psychological contract exhibit higher organizational citizenship behaviors (Hui et al., 2004; Shih and Chen, 2011; Li and Yu, 2017), improved performance in the organization (Ntalianis and Dyer, 2021), and low turnover rates (Umar and Ringim, 2015).

Finally, the presence of agreeable personality in new employees can increase the influence of mentoring on proactive behavior, balanced psychological contract. Agreeableness is one of the Big Five personality traits; nonetheless, few researchers have studied it, especially in organizational contexts. Although personality traits are heritable and relatively stable, they can change with factors such as environmental influences (Briley and Tucker-Drob, 2014). Therefore, organizations should control vicious competition and workplace bullying within the organization. These adverse organizational environments may lead to certain changes in the values of new employees, which may lead to changes in agreeable personality or a low state. Therefore, this study contributes to the theoretical and empirical research on agreeableness. It shows that mentoring can significantly impact new employees who have a high level of agreeableness, and can induce more proactive behaviors and a balanced psychological contract state. On this basis, the agreeable personality possessed by new employees also moderates the mediation model of balanced psychological contract. When new employees apply their agreeable personality traits in organizational work, they are more likely to perceive various forms of support from organizational mentoring due to the influence of their personality traits. This will lead them to fulfill their corresponding psychological contract, thereby generating positive proactive behaviors. This research result further validates the E (individual difference) × F line in the proactive motivation model proposed by Parker et al. (2010), which is a motivational mechanism that drives proactive behavior by stimulating individuals to be energized to act.

## Implications

### Theoretical contribution

Firstly, This research extends the proactive motivation model by Parker et al. (2010) with novel insights into the dynamics of mentoring, balanced psychological contracts, and agreeableness within the context of proactive behavior in organizational settings. A key innovation of this study lies in its exploration of how mentoring acts as a catalyst for proactive goal generation and striving, enriched by the mediating role of balanced psychological contracts and the moderating influence of agreeableness. Contrary to previous studies that primarily focused on direct influences, this research delineates a complex interplay between these factors, thereby offering a more granular understanding of the pathways leading to proactive behavior.

Secondly, this study connects the enterprise mentoring with the proactive behavior of new employees who are newly employed in the society, which provides some theoretical reference for the two research fields. This study not only discusses the influence of mentoring on proactive behavior, but also discusses its internal mediating mechanism and moderating mechanism, which enriches the theoretical support of employees’ proactive behavior in enterprise organizations.

Furthermore, this study contributes to the literature by examining the dual nature of agreeableness, acknowledging its potential drawbacks, such as excessive complacency or avoidance of necessary conflict, and how these might interact with cultural nuances in a Chinese context. This consideration introduces a critical perspective on the universal applicability of psychological theories, urging a cultural contingency approach in future research.

### Practical implications

Firstly, mentoring can improve the proactive behavior of new employees by providing new employees with a sense of security and dependence, similar to parents, through three major functions: career support, psychological support, and role modeling. This sense of security and dependence is a precondition for new employees to develop proactive motivation. These conditions can mitigate the negative effects of proactive behavior, thus enabling new employees’ total commitment to productive work behavior. Security also impacts individuals’ motivation to accomplish goals (Elliot and Reis, 2003; Levine and Heller, 2012). In a Chinese context, where societal values emphasize harmony, collectivism, and respect for authority, the manifestations and implications of agreeableness may differ from those in more individualistic cultures. For example, the positive aspects of agreeableness might be particularly valued and encouraged in China, aligning with the cultural emphasis on maintaining social harmony. However, the potential downsides, such as the risk of submissiveness or lack of assertiveness, may also be more pronounced or interpreted differently within this cultural framework. Organizations should design mentoring programs that not only aim to develop skills but also focus on enhancing the psychological well-being of new employees. This includes training mentors to recognize and cultivate not just agreeableness but also a balanced assertiveness in mentees, ensuring they can navigate workplace dynamics effectively.

Secondly, a simple dynamic psychological contract exists between new employees and the organization (Rousseau, 1995). The existing psychological contractual framework changes as new employees gain organizational experience (Sutton and Griffin, 2004; De Vos et al., 2009). Experience enables employees to provide feedback to the organization (O’Donohue et al., 2018), and the mentoring provides them with a better organizational experience by offering support professionally, psychologically, and in terms of role development, thereby benefiting their careers. At this time, a new employee will show a higher level of potential balanced psychological contract state to balance their relationship with the organization. Therefore, with the support of mentoring, a new employee’s balanced contract will show a higher-level state, leading to their proactive behavior toward the organization.

## Limitations and future research

This study, however, is not devoid of certain limitations. Firstly, all variables in this research were derived from individual self-reports, which indeed possess intrinsic merits and reflective authenticity. However, given the inherent human inclination towards self-enhancement, it is prudent to acknowledge the potential for response bias inherent in self-reporting. Furthermore, owing to a confluence of factors, encompassing the prevailing financial constraints and intricacies of interpersonal dynamics, the envisaged cross-level data collection involving both organizational and individual perspectives could not be fully realized within the scope of this study. Secondly, it remains plausible that the suitability of foreign-based measurement scales within the Chinese sociocultural milieu warrants scrutiny, and diligent assessment through a more expansive dataset is imperative. Subsequent research endeavors should contemplate the development of contextually pertinent measurement instruments specific to the Chinese milieu, thereby fostering a more nuanced understanding.

Furthermore, the prospect of extending the analytical purview beyond individual and team dimensions to encompass organizational contexts merits contemplation in future investigations. This comprehensive vantage could potentially unveil the intricate mechanisms underpinning proactive behavior, offering a more holistic comprehension of its multifaceted dynamics. For example: (1) to explore how each of the three functions of mentoring affects proactive behavior respectively; and (2) within the contexts of the ‘can do’ and ‘reason to’ pathways, to explore how the impact of other individual differences (such as values, accountability, self-beliefs, goal orientation, etc.) on the proactive behavior of new employees, and further investigate their interactive effects on factors influencing proactive behavior.

## Data availability statement

The original contributions presented in the study are included in the article/supplementary material, further inquiries can be directed to the corresponding authors.

## Ethics statement

The studies involving humans were approved by Gachon University Institutional Review Board. The studies were conducted in accordance with the local legislation and institutional requirements. Written informed consent for participation was not required from the participants or the participants’ legal guardians/next of kin in accordance with the national legislation and institutional requirements. Written informed consent was obtained from the individual(s) for the publication of any potentially identifiable images or data included in this article.

## Author contributions

WY: Funding acquisition, Investigation, Methodology, Writing – original draft. YW: Data curation, Formal analysis, Resources, Writing – review & editing. MC: Conceptualization, Formal analysis, Supervision, Writing – review & editing. YL: Methodology, Software, Validation, Writing – review & editing.
